# Alpha-fetoprotein-elevated postpubertal testicular teratoma with retroperitoneal metastasis on ^18^F-FDG PET/CT: case report and literature review

**DOI:** 10.3389/fmed.2023.1269587

**Published:** 2023-09-05

**Authors:** Hao Jiao, Yongkang Qiu, Wenpeng Huang, Yongbai Zhang, Zhao Chen, Aixiang Wang, Lei Kang

**Affiliations:** ^1^Department of Nuclear Medicine, Peking University First Hospital, Beijing, China; ^2^Department of Urology, Peking University First Hospital, Institute of Urology, Peking University, National Urological Cancer Center, Beijing, China

**Keywords:** postpubertal testicular teratoma, retroperitoneal metastasis, ^18^F-FDG, PET/CT, alpha-fetoprotein

## Abstract

Postpubertal testicular teratoma exhibits malignant biological behavior and has metastatic potential. We report a case of a 17-year-old patient diagnosed with postpubertal testicular teratoma with massive retroperitoneal metastasis. The pathological examination revealed a mature teratoma without any other components. However, the patient had a significantly increased level of AFP, and ^18^F-FDG PET/CT showed the retroperitoneal metastasis had increased FDG uptake, with a SUVmax of 15.6, suggesting the coexistence of other germ cell tumor components, and the patient might have a poor prognosis. After resection of the retroperitoneal tumor, PET/CT further revealed multiple abdominal and pelvic metastases, with a SUVmax of 22.5. Therefore, the patient received a cycle of chemotherapy and follow-up PET/CT imaging showed the achievement of complete metabolic response after the treatment. In this case, PET/CT played a crucial role in detecting metastasis, compensating for the limitations of pathological sampling, thus establishing a definitive diagnosis and predicting prognosis. And it was evident that PET/CT also has the advantage of evaluating therapeutic efficacy.

## Introduction

The World Health Organization updated the classification of testicular tumors in the “Tumors of the urinary system and male genital organs” category in 2022, among which nonseminomatous germ cell tumors (NSGCTs) include embryonal carcinomas, yolk sac tumors, choriocarcinomas, and teratomas. Testicular teratomas can be further classified into prepubertal and postpubertal teratomas, and teratomas unrelated to germ cell neoplasia *in situ* (GCNIS) are designated as prepubertal teratomas, while the GCNIS-derived entities are designated as postpubertal type (1, 2). Prepubertal testicular teratomas typically occur in children and exhibit a benign biological behavior. On the other hand, postpubertal testicular teratomas usually occur in adults and may also occur in pediatric patients who have disorders of sex development (3). The diagnosis of teratomas relies on pathological examination. Postpubertal teratomas, whether mature or immature teratomas, are considered as malignant components and have metastatic potential (4, 5). A significant elevation of serum alpha-fetoprotein (AFP) suggests the potential coexistence of yolk sac tumor components in the teratoma (6). However, due to sampling limitations, pathological examination failed to show the presence of yolk sac tumors. ^18^F-fluorodeoxygluxose (FDG) positron emission tomography/computed tomography (PET/CT) is used as a favorable examination to detect systemic tumor metastasis. Mature teratomas have normal or slightly elevated FDG uptake, so if the tumor has a significantly elevated FDG uptake, it suggests that there are other components in the tumor (7, 8). Combined with high FDG uptake on PET/CT images and significantly increased AFP, patients can be diagnosed more accurately. And a patient with teratoma coexisting with yolk sac tumor components usually has a poor prognosis, PET/CT can play a role in detecting metastasis and evaluating treatment efficacy (9).

## Case presentation

**Figure 1:**
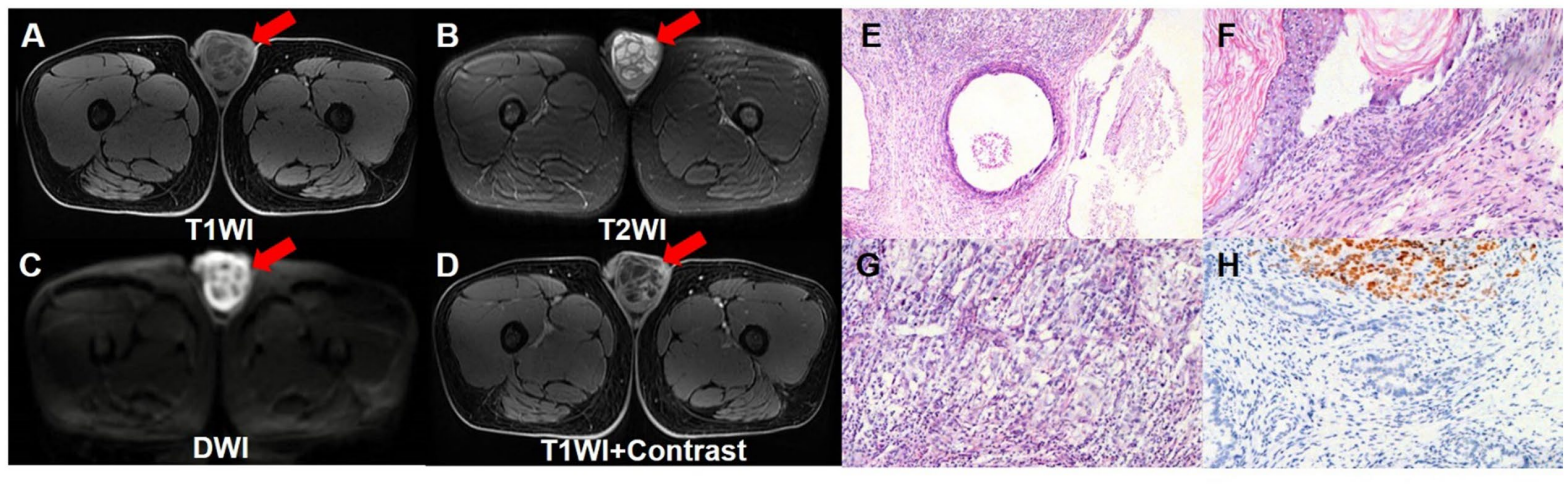
Pelvic contrast-enhanced MRI images and pathological images of the testicular teratoma. Pelvic contrast-enhanced MRI revealed that the left testicular mass contained both solid and cystic components, and it exhibited low signal intensity on T1WI transverse image **(A)**, and high signal intensity on T2WI transverse image **(B)**. There were multiple reticular structures in the mass, which exhibited low signal intensity on T1WI and T2WI, and exhibited high signal intensity on DWI transverse image **(C)**. On enhancement scan image **(D)**, the solid components showed significant enhancement. **(E–G)** The hematoxylin–eosin (HE) staining (magnification ×40 and 100) showed that the mature teratoma was infiltrated with mostly well-differentiated tissues, while focal areas showed markedly heterotypic epithelioid cells. **(H)** Immunohistochemistry showed that the tumor cells were positive for SALL4 (magnification ×100).

A 17-year-old boy accidentally found a left testicular swelling, which was hard and accompanied by pain. The patient went to the local hospital and was diagnosed as inflammation. The pain was relieved after antibiotic infusion, but there was no significant change in the size of the swelling, so he was consulted at our hospital. The patient was previously in good health and had no history of tumor, and had no familial history of hereditary diseases. Laboratory tests showed markedly increased level of AFP (>1,210 ng/mL) whereas his HCG level was normal (<0.1 mIU/mL). The ultrasound showed that there was a cystic mass in the left testis, which tended to be benign. The pelvic contrast-enhanced MRI revealed that the left testicular mass contained both solid and cystic components, and it exhibited low signal intensity on T1WI and high signal intensity on T2WI. There were multiple reticular structures in the mass, which exhibited low signal intensity on T1WI and T2WI and high signal intensity on DWI. The solid components showed significant enhancement on enhancement scan. And there were no enlarged lymph nodes in the scanning range (Figures 1A–D). Then, left side radical orchiectomy was performed. The resected specimen measured 5.5 × 4.5 × 3.3 cm, and the pathological examination revealed a mature teratoma, with mostly well-differentiated tissues, while focal areas showed markedly heterotypic epithelioid cells. Immunohistochemistry staining results demonstrated the positive expression of AE1/AE3, SALL4, CK7 and Ki-67 (20%), whereas the negative expression of CD30, EMA, CEA, PLAP, AFP2, Vim, CD117, S-100, TTF1, PSA, CK20 (Figures 1E–H).

**Figure 2:**
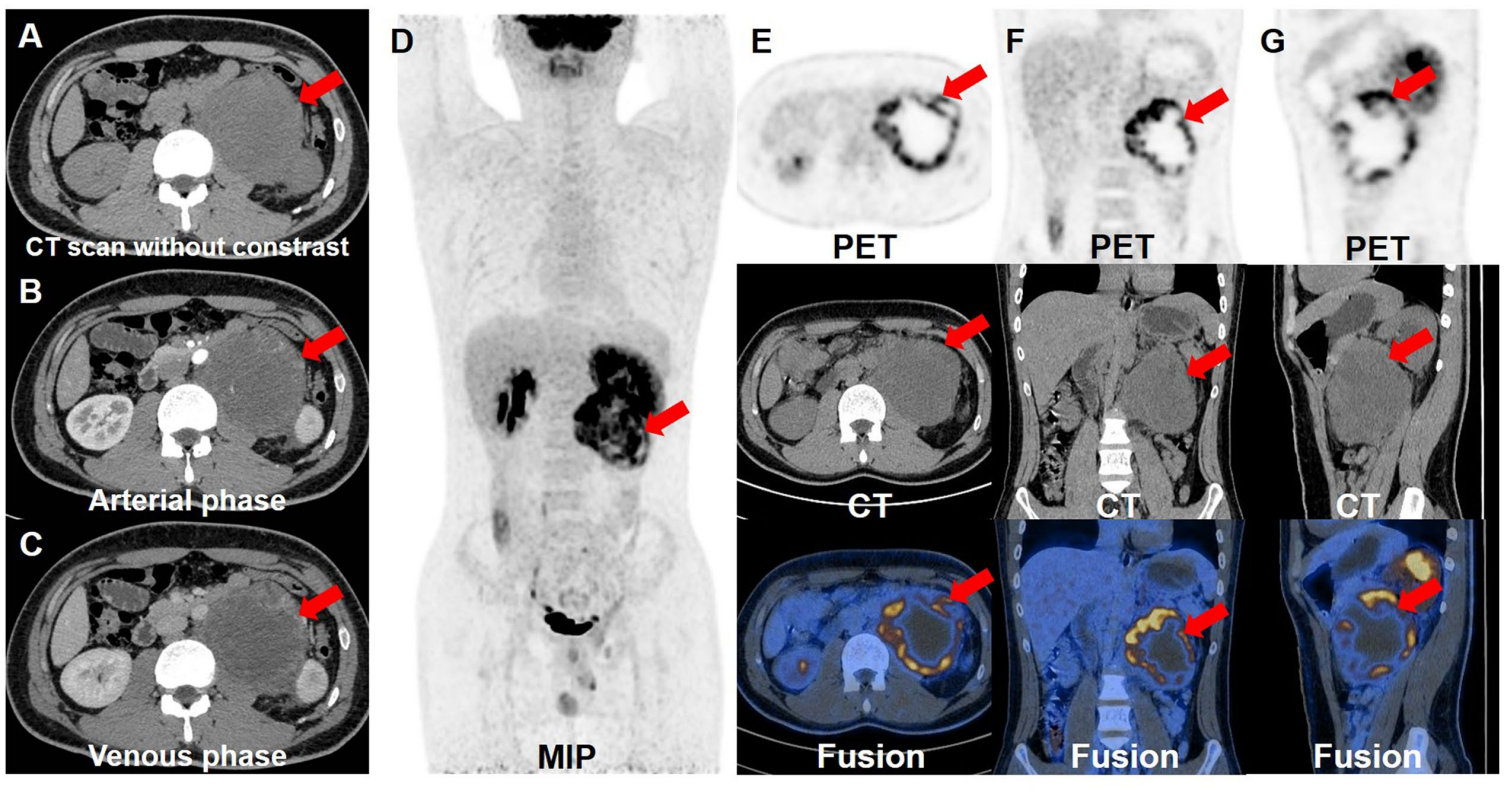
Contrast-enhanced CT and ^18^F-FDG PET/CT images. The retroperitoneal teratoma exhibited an incomplete capsule and unclear local boundaries on the enhanced CT plain scan **(A)**, with uneven internal density. And it demonstrated uneven enhancement during the arterial **(B)** and venous **(C)** phases of the contrast-enhanced CT. **(D)** The whole-body maximum intensity projection showed the retroperitoneal lesion with hypermetabolism. The transverse **(E)**, coronal **(F)** and sagittal **(G)** images of retroperitoneal lesions showed that the tumor had high FDG uptake, with a SUVmax of 15.9.

However, half a month after the excision of testicular lesion, although the patient had no clinical symptoms, the serum level of AFP remained significantly increased (>1,210 ng/mL), so further examinations were performed. Contrast-enhanced CT of urinary system showed there was an irregular soft tissue mass on the on the left side of abdominal aorta and below the left renal hilum, at the size of 8.4 × 10.0 cm. The mass had an incomplete capsule with unclear boundary. The internal density was heterogeneous and multiple patchy necrotic areas could be found. Also, the fatty space surrounding the lesion was unclear and the lesion demonstrated uneven enhancement (Figures 2A–C). To further evaluate the whole-body situation and re-staging, ^18^F-FDG PET/CT was performed. PET/CT revealed increased FDG uptake at the edges of the tumor with a SUVmax of 15.9, while the central FDG uptake was low, indicating a large area of necrosis. These findings reflected the aggressive growth and high malignant potential of the tumor. In addition, there were no metastatic lesions with abnormal FDG uptake in other parts of the body (Figures 2D–G). Then, the patient underwent resection of the retroperitoneal tumor, retroperitoneal lymphadenectomy and nephrectomy of the left side. Postoperative pathological examination of the resected specimen also revealed a mature teratoma with no other components. Immunohistochemistry staining results demonstrated the positive expression of AE1/AE3, CK7, AFP and Ki-67 (30%), whereas the negative expression of CD30, OCT3/4, CK20 and p53. Based on pathological findings, postpubertal testicular teratoma should be considered and the final diagnosis for the patient was mature teratoma of the testis with retroperitoneal metastasis.

**Figure 3:**
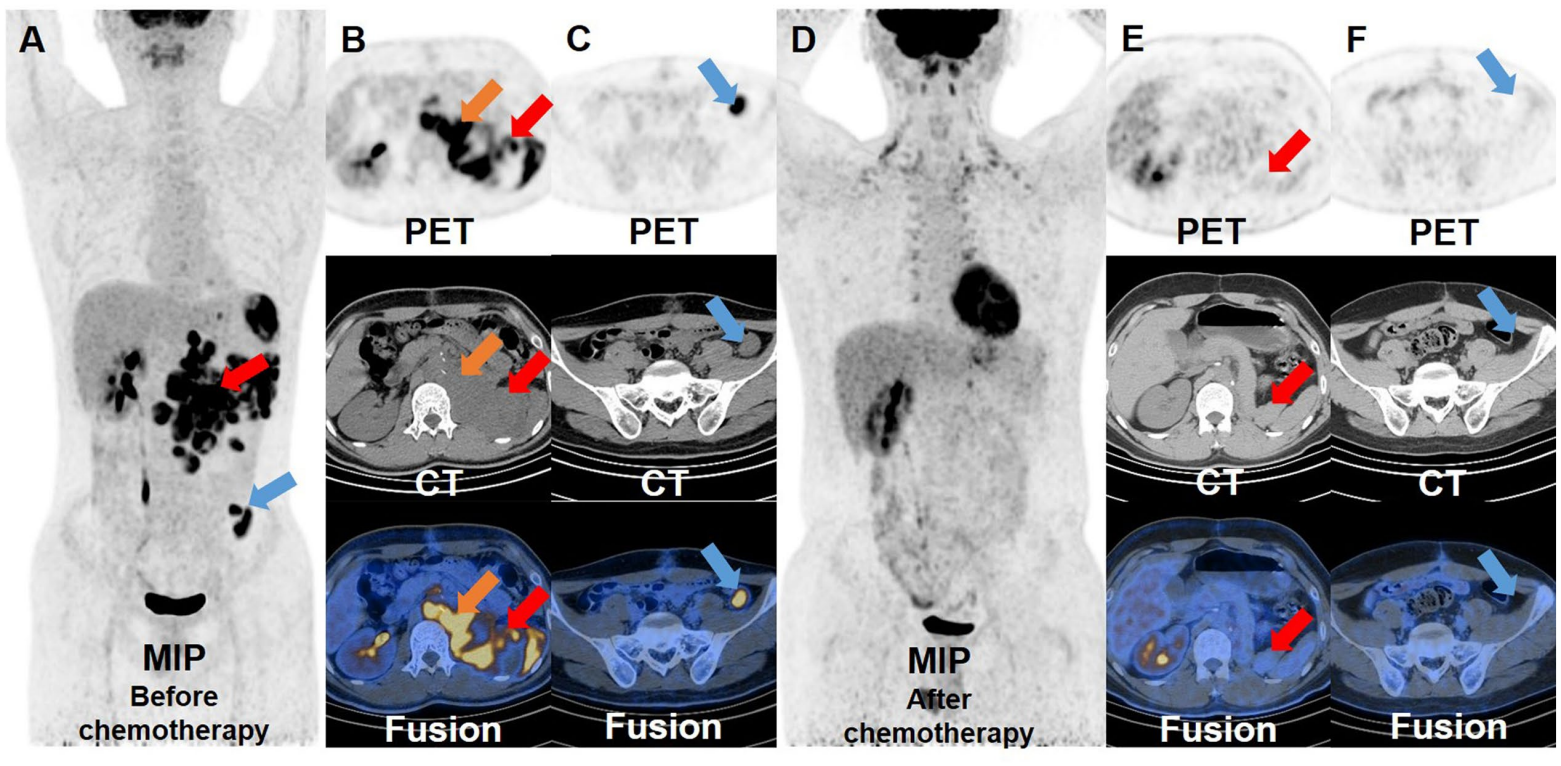
^18^F-FDG PET/CT images before and after chemotherapy. Before the patient received chemotherapy, the whole-body maximum intensity projection **(A)** showed multiple hypermetabolic lesions around the abdominal aorta, spleen, left psoas major, left diaphragm foot and left pelvic cavity. And the transverse images **(B,C)** showed that these lesions had significantly increased FDG uptake, with a SUVmax of 22.5. After the patient received a cycle of chemotherapy, the whole-body maximum intensity projection **(D)** and the transverse images **(E,F)** showed that the pre-existing lesions shrank or disappeared, and the remaining lesions displayed a marked reduction in FDG uptake compared to before.

One month after the second operation, the patient’s AFP was still higher than normal (>1,210 ng/mL), so he underwent PET/CT examination again for re-staging. There were multiple soft tissue density nodules and masses around the abdominal aorta, spleen, left psoas major, left diaphragm foot and left pelvic cavity, some of which had unclear boundaries with adjacent intestines, left adrenal gland and psoas major muscle. The FDG uptake of these lesions increased unevenly, with a SUVmax of 22.5, indicating extensive recurrent metastasis of the tumor (Figures 3A–C). Then, the patients received one cycle of combination chemotherapy with bleomycin, etoposide and cisplatin. In half a year, the patient underwent PET/CT examinations twice to monitor the effect of treatment. Three months after chemotherapy, PET/CT showed some lesions shrank or disappeared to varying degrees, and FDG uptake was lower than before with a SUVmax of 8.4. Furthermore, the PET/CT results after chemotherapy showed that most of the lesions became smaller or disappeared, and FDG uptake became normal, achieving complete metabolic response recovery and suggesting that the treatment was effective (Figures 3D–F).

## Discussion

Although testicular tumors account for only about 1% of all solid tumors in men, it is the most common solid malignancy in men aged 15–35 years (10). The World Health Organization updated the classification of testicular tumors in the “Tumors of the urinary system and male genital organs “category in 2022 (1). The two main types of testicular tumors are germ cell tumors (GCTs) and sex cord-stromal tumors (SCSTs), with the former accounting for 95% of cases. GCTs can be divided into pure seminomas, which consist entirely of germ cells, and all other tumors collectively called NSGCTs. Teratomas, along with embryonal carcinomas, yolk sac tumors, and choriocarcinomas, are classified as NSGCTs. Testicular teratoma can be classified into prepubertal and postpubertal teratoma. Prepubertal teratoma usually occurs in children and is typically a pure teratoma with benign biological behavior (11). However, postpubertal testicular teratomas typically occur in adults and are rarely pure teratomas. They exhibit malignant biological behavior and 22–38% of patients develop metastases (4, 5). Only a minority of teratomas contain malignant somatic tissue components, such as squamous cell carcinoma, adenocarcinoma, immature neuroectodermal elements, or sarcoma (12, 13). Testicular teratomas are characterized by slow progression and lack of typical clinical presentation, presenting with testicular enlargement, heaviness, discomfort, and mild pain. If a teratoma exhibits malignant biological behavior, the patient may present with rapid enlargement of the tumor, as well as systemic symptoms such as anemia and weight loss. If the tumor metastasizes, the patient may also experience other clinical symptoms such as progressive abdominal distension and disturbed bowel habits (14). Most patients with retroperitoneal teratoma are asymptomatic, and the most common symptom is nonspecific pain. Patients whose adrenals were compressed may present with vertigo and hypertension (15). In our case, the patient’s testicular teratoma caused pain, while the retroperitoneal teratoma did not cause any symptoms, which was consistent with previous studies.

Various imaging modalities such as ultrasound, MRI and CT are used for evaluating testicular teratomas. Ultrasound evaluation is often the first choice of imaging modality for testicular teratoma, which typically shows a cystic or solid cystic mass with heterogeneous echogenicity. The mass may contain calcifications and other elements, such as bone and hair. Immature teratomas are richly vascularized due to their predominantly solid component, while mature teratomas are mainly cystic and lack vascularity (16, 17). When a teratoma undergoes malignant somatic transformation, it may initially present as a cystic lesion with low-level echogenicity and gradually become solid, with a heterogeneous pattern of echogenicity and rich blood flow (18). A benign teratoma typically presents as an ellipsoid mass with distinct borders on MRI. Mixed signal intensity is observed on both T1WI and T2WI, and the presence of high signal intensity fat can also be detected. Contrast-enhanced scanning shows mild and heterogeneous enhancement. Meanwhile, a malignant teratoma can present with an indistinct border and heterogeneous signal intensity, with significant and heterogeneous enhancement (19). CT has a limitation in evaluating soft tissue components, but it still widely used for patients with teratomas because it is superior to MRI in scanning time, cost and scanning coverage. The typical CT manifestation of teratoma consists of a heterogeneous cystic-solid mass composed of various components, including fat, hair, and fluid. The cyst wall can vary in thickness and may exhibit arc-shaped calcification. The density of the fat component within the cyst is negative. On contrast-enhanced scans, the solid portion and the septa of the mass show mild to moderate enhancement, while the low-density areas and the fat components remain unenhanced. Malignant teratoma is characterized by the presence of a nodular forming and enhancing soft tissue component, extracapsular tumor growth with extension into adjacent structures or metastasis, etc. (20).

The final diagnosis of testicular teratoma relies on pathological examination. Mature teratomas contain well-differentiated tissues, typically including three germ layers. Immature teratomas refer to tumors containing undifferentiated components, resembling tissues at embryonic developmental stage, characterized by immature neuroepithelial islands and varying degree of differentiation including skeletal muscle, cartilage, and stromal cells. For patients with testicular GCT, the distinction between mature and immature teratoma is not of great significance, because both are considered as malignant components and have metastatic potential. Approximately one-third of testicular GCTs are mixed, presenting with two or more different GCT types. Teratoma can coexist with embryonal carcinoma and yolk sac tumor (21, 22). A small number of teratomas exhibit somatic malignant transformation, and pathological examination can reveal that these tumors contain squamous cell carcinoma, adenocarcinoma, immature neuroectodermal components, or sarcomatous components (23).

Considering that postpubertal testicular teratomas have metastatic potential, ^18^F-FDG PET/CT examination combined with morphological and metabolic information can be used as a favorable examination to detect metastasis. A literature search was performed on the PubMed and database from 1997 to 2022, using the keyword “retroperitoneal teratoma” and “PET/CT.” There were night cases of retroperitoneal teratoma with PET/CT findings. The basic information and metabolism of retroperitoneal teratoma are summarized in Table 1 (15, 24–29). Out of these night cases, only one patient was 7 months old, while other patients were adults, ranging in age from 23 to 49 years, with more males than females. Consistent with most reports, the patient we reported was a near-adult male. Retroperitoneal teratoma generally did not cause clinical symptoms, as reported in our case, but might also presented as abdominal pain. When the tumor invaded the spine, patients might present with back pain. When the tumor was a pure mature teratoma, there was usually no increased FDG uptake or slightly increased FDG uptake, which was consistent with previous findings that mature teratomas had a very low avidity for ^18^F-FDG, with a SUVmean of 1.38 (7, 8). However, one infant with a mature teratoma had a SUVmax of 4.1, possibly due to the abundant central nervous tissue (24). In addition to this, if mature teratomas showed increased FDG uptake, possibly due to the presence of immature teratoma, embryonal carcinoma, or malignant somatic tissue components. However, none of these components were found in the pathological examination of the patient in our case. It is also worth mentioning that among the above adult patients, patients with pure mature teratoma, or teratoma combined with embryonal carcinoma, or teratoma combined with malignant somatic transformation all had normal levels of AFP (<7.5 ng/mL), whereas the patient in our case had a significantly increased AFP level (>1,210 ng/mL). Literature reports suggested that elevated AFP or β-HCG could not be attributed to teratoma components, and an increase in these tumor markers suggested the presence of coexisting other GCT components (22, 30). For example, significant elevation of AFP indicated the presence of yolk sac tumor components in the tumor (6). The pathological examination of this case did not reveal the presence of yolk sac tumor components, possibly due to the limited sampling site. However, combining the significantly elevated AFP level and the hypermetabolism of the tumor on PET/CT images, it can be inferred with high probability that this patient had a mixed GCT, more specifically, a combination of teratoma and yolk sac tumor. SALL4 is a transcription factor and serves as a marker of yolk sac tumor (31, 32). In order to verify the presence of yolk sac tumor components in the patient’s primary testicular teratoma, we reperformed immunohistochemical staining of the testicular teratoma pathology images and found that the tumor cells were positive for SALL4, suggesting that there was a high probability of the presence of yolk sac tumor components in the lesion. In terms of prognosis, it has been reported that patients had a poor prognosis if other GCT components were present in teratomas, especially in mature teratomas (9). In addition, AFP levels were correlated with disease severity (30). Therefore, the patient might have a very poor prognosis. In our case, ^18^F-FDG PET/CT played an important role in patients with testicular teratoma and metastatic retroperitoneal teratoma, as it could compensate for the limitations of pathological sampling, clarify the diagnosis, and provide more accurate information about the prognosis.

**Table 1:** Literature review of retroperitoneal teratoma cases with ^18^F-FDG PET/CT manifestations.

No.	References	Age	Sex	Clinical symptoms	Primary tumor site	AFP	Pathological type of the retroperitoneal tumor	^18^F-FDG uptake of the retroperitoneal tumor
1	Suh et al. (24)	7 months	F	Abdominal distention	Retroperitoneum	27.17 ng/mL	Mature teratoma	Slight increase (SUVmax 4.1)
2	Ma et al. (15)	40 years	F	Asymptomatic	Retroperitoneum	Normal (<7.5 ng/mL)	Dermoid cyst (a kind of mature cystic teratoma)	Slight increase
3	Quak et al. (25)	41 years	M	Asymptomatic	Testis	Normal (<7.5 ng/mL)	Mature teratoma with malignant somatic transformation	Significant increase
4	Rodrigo et al. (26)	42 years	M	Not mentioned	Testis	Normal (<7.5 ng/mL)	Teratoma with malignant somatic transformation	Significant increase (SUVmax 14.7)
5	Rodrigo et al. (26)	47 years	M	Not mentioned	Testis	Normal (<7.5 ng/mL)	Teratoma with malignant somatic transformation	Slight increase (SUVmax 5)
6	Yousef et al. (27)	49 years	M	Not mentioned	Testis	Normal (<7.5 ng/mL)	Mature teratoma	Slight increase
7	Martin et al. (28)	23 years	M	Not mentioned	Testis	Normal (<7.5 ng/mL)	Mature teratoma	Normal
8	Daniel et al. (29)	33 years	M	Abdominal pain	Testis	Normal (<7.5 ng/mL)	Teratoma with embryonal carcinoma	Significant increase
9	Daniel et al. (29)	38 years	M	Back pain	Testis	Normal (<7.5 ng/mL)	Mature and immature teratomas with minimal viable malignant tumor	Significant increase

The standard treatment for all GCTs in adults is radical orchiectomy. Patients with postpubertal testicular teratoma have a high incidence of retroperitoneal metastasis, with rates of 30% in patients with clinical stage I and 75% in patients with clinical stage II (33). Therefore, the patient underwent imaging examination of the retroperitoneum, which revealed a massive retroperitoneal metastatic teratoma. Studies have shown that even in patients with pure testicular teratoma, approximately 20% of patients may experience recurrence during surveillance (34, 35). Thus, after undergoing retroperitoneal tumor resection, the patient received regular PET/CT scans to monitor tumor recurrence. One month after surgery, PET/CT revealed extensive recurrent lesions with high FDG uptake, with a SUVmax of 22.5. The patient then underwent chemotherapy and also used PET/CT to test the efficacy of the treatment. Late relapse is rare in patients with testicular GCTs, and follow-up may not be necessary to detect recurrence after 5 years (36). However, this patient not only presented with retroperitoneal metastasis but also had extensive recurrent lesion with hypermetabolism in the abdominal and pelvic cavity revealed by PET/CT, indicating the need for a longer follow-up period.

## Conclusion

Postpubertal testicular teratoma has metastatic potential and ^18^F-FDG PET/CT can be used as a favorable examination to detect systemic tumor metastasis. In addition, although the diagnosis of teratoma relies on pathological examination, it can be inaccurate due to sampling limitations. If AFP levels are significantly elevated and PET/CT shows lesions with high FDG uptake, the presence of yolk sac tumor component in the teratoma should be suspected. PET/CT helps to compensate for the limitations of pathological sampling, thus confirming the diagnosis. If teratoma coexists with other GCT components, the patient has a poorer prognosis, and PET/CT plays an important role in predicting patient prognosis, monitoring tumor metastasis and recurrence, and evaluating the therapeutic effect.

## Data availability statement

The original contributions presented in the study are included in the article/supplementary material, further inquiries can be directed to the corresponding authors.

## Ethics statement

Written informed consent was obtained from the minor(s)’ legal guardian/next of kin for the publication of any potentially identifiable images or data included in this article.

## Author contributions

HJ: Writing – original draft, Writing – review & editing. YQ: Writing – original draft, Writing – review & editing. WH: Writing – review & editing. YZ: Writing – review & editing. ZC: Writing – review & editing. AW: Writing – review & editing, Supervision. LK: Writing – review & editing, Supervision.

## Funding

The author(s) declare financial support was received for the research, authorship, and/or publication of this article.

This work was supported by the National Natural Science Foundation of China (82171970), the Beijing Science Foundation for Distinguished Young Scholars (JQ21025), the Beijing Municipal Science & Technology Commission (Z221100007422027), National High Level Hospital Clinical Research Funding (Interdisciplinary Research Project of Peking University First Hospital, 2023IR17).

## Conflict of interest

The authors declare that the research was conducted in the absence of any commercial or financial relationships that could be construed as a potential conflict of interest.

## Publisher’s note

All claims expressed in this article are solely those of the authors and do not necessarily represent those of their affiliated organizations, or those of the publisher, the editors and the reviewers. Any product that may be evaluated in this article, or claim that may be made by its manufacturer, is not guaranteed or endorsed by the publisher.
